# Effect of tranexamic acid by baseline risk of death in acute bleeding patients: a meta-analysis of individual patient-level data from 28 333 patients

**DOI:** 10.1016/j.bja.2020.01.020

**Published:** 2020-03-19

**Authors:** Francois-Xavier Ageron, Angele Gayet-Ageron, Katharine Ker, Timothy J. Coats, Haleema Shakur-Still, Ian Roberts, for the Antifibrinolytics Trials Collaboration, Aasia Kayani, Amber Geer, Bernard Ndungu, Bukola Fawole, Catherine Gilliam, Cecelia Adetayo, Collette Barrow, Danielle Beaumont, Danielle Prowse, David I'Anson, Eni Balogun, Hakim Miah, Haleema Shakur-Still, Ian Roberts, Imogen Brooks, Julio Onandia, Katharine Ker, Kiran Javaid, Laura Suncuan, Lauren Frimley, Mia Reid, Monica Arribas, Myriam Benyahia, Olujide Okunade, Phil Edwards, Rizwana Chaudhri, Sergey Kostrov, Sneha Kansagra, Tracey Pepple

**Affiliations:** 1 Clinical Trials Unit, London School of Hygiene and Tropical Medicine, London, UK; 2 Emergency Department, Lausanne University Hospital, CHUV, Lausanne, Switzerland; 3 Division of Clinical Epidemiology, University Hospitals of Geneva, Geneva, Switzerland; 4 Emergency Medicine, University of Leicester, Leicester, UK

**Keywords:** antifibrinolytics, bleeding, coagulopathy, mortality, postpartum haemorrhage, trauma

## Abstract

**Background:**

Early administration of the antifibrinolytic drug tranexamic acid reduces death from bleeding in trauma and postpartum haemorrhage. We examined how the effectiveness and safety of antifibrinolytic drugs varies by the baseline risk of death as a result of bleeding.

**Methods:**

We performed an individual patient-level data meta-analysis of randomised trials including more than 1000 patients that assessed antifibrinolytics in acute severe bleeding. We identified trials performed between January 1, 1946 and July 5, 2018 (PROSPERO, number 42016052155).

**Results:**

Two randomised trials were selected where 28 333 patients received tranexamic acid treatment within 3 h after the onset of acute bleeding. Baseline characteristics to estimate the risk of death as a result of bleeding were divided into four categories: Low (0–5%), intermediate (6–10%), high (11–20%), and very high (>20%). Most patients had a low baseline risk of death as a result of bleeding (23 008 [81%]). Deaths as a result of bleeding occurred in all baseline risk categories with 240 (1%), 202 (8%), 232 (14%), and 357 (30%) deaths in the low-, intermediate-, high-, and very high-risk categories, respectively. The effectiveness of tranexamic acid did not vary by baseline risk when given within 3 h after bleeding onset (*P*=0.51 for interaction term). There was no increased risk of vascular occlusive events with tranexamic acid and it did not vary by baseline risk categories (*P*=0.25).

**Conclusions:**

Tranexamic acid appears to be safe and effective regardless of baseline risk of death. Because many deaths are in patients at low and intermediate risk, tranexamic acid use should not be restricted to the most severely injured or bleeding patients.

**Editor's key points**

• This meta-analysis investigated how the effectiveness and safety of tranexamic acid varies by the baseline risk of death as a result of acute bleeding.
• The study shows that many deaths from bleeding are in patients at low or intermediate risk.
• The effectiveness of tranexamic acid seems not to vary by the baseline risk of patients.
• Tranexamic acid should therefore not be limited to the most severely injured or bleeding patients.

The Anti-Fibrinolytic Trials Collaboration previously published a meta-analysis of individual patient data showing that early administration of tranexamic acid safely reduces death from acute severe bleeding.^1^ When given soon after bleeding onset, tranexamic acid reduces the relative risk of death as a result of bleeding by about one-third. Early tranexamic acid treatment is widely recommended in treatment guidelines for acute severe bleeding, including postpartum haemorrhage and major trauma.^2^, ^3^, ^4^

Many guidelines, especially those for trauma, focus on the use of tranexamic acid in severely injured patients with a high risk of death from bleeding.^5^^,^^6^ Although these patients have much to gain from tranexamic acid treatment, they are few in number and many die at the scene.^7^ Because there are many more patients with less severe injuries and a lower risk of death from bleeding, if tranexamic acid was similarly effective, prompt treatment of these patients could prevent many deaths. We examined how the effectiveness and safety of antifibrinolytic drugs vary by the baseline risk of death as a result of bleeding.

## Methods

### Design and selection criteria

We conducted an individual patient data meta-analysis of randomised, placebo-controlled trials conducted between January 1, 1946 and July 5, 2018. The methods and the selection criteria were described previously.^1^ The study protocol was registered in November 2016 (PROSPERO, number 42016052155).^8^ Any randomised trial with more than 1000 patients that assessed the effects of antifibrinolytic drugs (aprotinin, tranexamic acid, aminocaproic acid, and aminomethylbenzoic acid) in patients with acute bleeding was eligible for inclusion. We identified trials from a permanent register of antifibrinolytic trials maintained by the London School of Hygiene and Tropical Medicine Clinical Trials Unit. The register is based on searches of MEDLINE, Embase, the Cochrane Central Register of Controlled Trials, Web of Science, PubMed, Popline, and the WHO International Clinical Trials Registry Platform (Supplementary file S1). Three reviewers (AG-A, KK, F-XA) independently extracted data. We selected trials recruiting patients with acute bleeding at the time of randomisation (treatment trials). We excluded patients who were randomised more than 3 h after bleeding onset, since previous studies have shown that antifibrinolytics are ineffective after this period. We prepared a statistical analysis plan before searching for trials. Patients and the public were not involved in the research.

### Outcome

The primary outcome was death as a result of bleeding. This is the most relevant primary outcome given the mechanism of action of antifibrinolytic drugs. All-cause mortality includes non-bleeding related deaths, such as sepsis, that should not be affected by antifibrinolytics. Because these deaths could dilute the treatment effect, important benefits or harms could be obscured in all-cause mortality.^9^ Moreover, because the relative contributions of non-bleeding deaths will vary between populations, all-cause mortality is not widely generalisable. Secondary outcomes were fatal and non-fatal vascular occlusive events (myocardial infarction, stroke, pulmonary embolism, and DVT).

### Data analysis

We evaluated the quality of included trials by assessing sequence generation, allocation concealment, blinding, data completeness, and risk of selective reporting. Analysis was based on individual patient-level data. We estimated the baseline risk of death as a result of bleeding separately for each trial. We used prognostic models to predict the baseline risk using multivariate logistic regression. We used a previously published prognostic model for trauma.^10^ Because there were no suitable prognostic models for postpartum haemorrhage, we used the same method to develop a prognostic model for postpartum haemorrhage. We only used baseline characteristics collected before randomisation as predictors. To improve the precision of our models, we included all trial participants from the treatment and placebo groups.^11^ We included all potential predictors at baseline and adjusted for the use of antifibrinolytic drugs. We included linear and polynomial terms for continuous variables. We used the backward stepwise method and removed one at a time, variables for which there was no evidence of association (*P*-value for the Wald test >0.05). To estimate the risk at baseline, the coefficient for antifibrinolytic drugs was constrained at 0 in the equation. We performed sensitivity analysis that estimated the baseline risk in the placebo arm and present the results in the supplementary files. The estimates would be less precise, but may avoid misclassification from assuming a constant effect of tranexamic acid. The predicted baseline risk of death as a result of bleeding was estimated for each trial participant in both treatment groups. For each prognostic model, we assessed the performance by estimating discrimination and calibration. Discrimination represents the ability of the model to identify a patient with the outcome of interest and is evaluated by the concordance statistic (C-Statistic). Calibration represents the agreement between predicted and observed risk. On the basis of the predicted baseline risk, participants were assigned to one of the four baseline categories of risk of death as a result of bleeding: 0–5% (low); 6–10% (intermediate); 11–20% (high), and >20% (very high). The categories were chosen because they were clinically relevant, easy to understand (using a base of 5 or 10), and consistent with previous studies.^12^^,^^13^

All analyses were done according to the intention-to-treat principle. We reported continuous variables as mean (standard deviation) and median (inter-quartile range). We reported categorical variables as numbers and proportions. We plotted frequency distributions for baseline risk in all participants and in patients who died from bleeding. We estimated the effect of antifibrinolytics on death as a result of bleeding within categories of baseline risk and provide crude risk ratios. We tested the homogeneity of treatment effect across these between categories of risk using the χ^2^ test. We used logistic regression to assess the effects of antifibrinolytics on death as a result of bleeding and reported treatment effects with odds ratios and 95% confidence interval (CI). First, we tested the homogeneity of the treatment effect between trials by including an interaction term between treatment and trial and reporting the *P*-value (model 1, Supplementary file S2). We hypothesised that the treatment effect does not vary by baseline risk, unlike time to treatment for which treatment delay reduces the treatment benefit.^1^ To verify the homogeneity of the effect of baseline risk on treatment effect by time to treatment, we performed a second model with a triple interaction between the terms for baseline risk, the treatment group, and the time to treatment (model 2, Supplementary file S2). Once the homogeneity of the treatment effect with baseline risk and time to treatment was verified, we ran a third model to assess the homogeneity between the treatment effect and baseline risk adjusting for trial and time to treatment (model 3, Supplementary file S2). We reported the *P*-value for the interaction term between treatment effect and baseline risk and plotted the treatment effects with odds ratios and 95% CI according to baseline risk.

### Missing values

There were no missing outcome data, but there were missing values for some predictor variables. In order to estimate baseline risks on the full dataset, we replaced missing predictors using multiple imputation with 20 imputed datasets and adjustment of the imputation model for death as a result of bleeding, age, systolic BP, ventilatory frequency, and Glasgow outcome scale.

## Results

**Fig 1:**
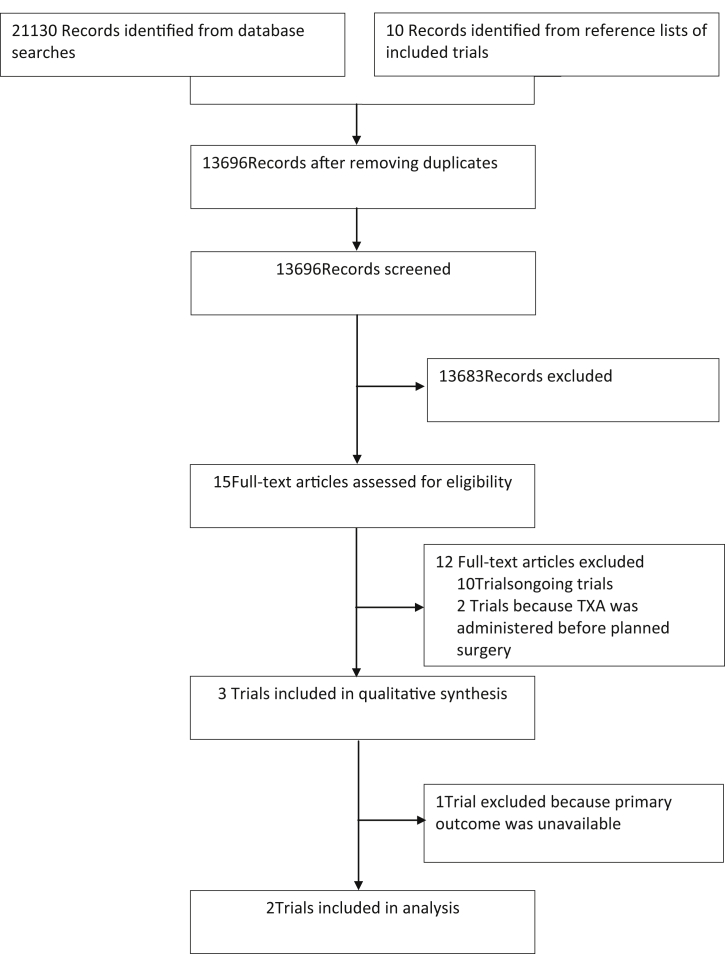
Study selection. TXA, tranexamic acid.

Figure 1 shows the number of records identified and the reasons for exclusions. We found five completed^14^, ^15^, ^16^, ^17^, ^18^ and 10 ongoing trials^19^, ^20^, ^21^, ^22^, ^23^, ^24^, ^25^, ^26^, ^27^, ^28^ (Supplementary file S3). All trials used tranexamic acid. Three trials met our inclusion criteria. The CRASH-2 and WOMAN trials received ethics committee approval from the London School of Hygiene and Tropical Medicine, UK and the ethics committees of all participating hospitals. The CRASH-2 trial included 20 211 trauma patients and assessed the effects of tranexamic acid on death and vascular occlusive events. Data from the CRASH-2 trial are available via freeBIRD (free bank of injury and emergency research data), hosted by the Clinical Trial Unit (CTU) of the London School of Hygiene and Tropical Medicine (https://ctu-app.lshtm.ac.uk/freebird). The WOMAN trial assessed the effects of tranexamic acid on death and serious morbidity in 20 060 women with postpartum haemorrhage. The TICH-2 trial assessed the effect of tranexamic acid on death and dependency in non-traumatic intracerebral haemorrhage. Exsanguination does not normally occur in adults with cerebral haemorrhage. Death usually arises as a result of cerebral injuries and high ICP. The TICH-2 trial was excluded from analysis as it was not possible to collect the primary outcome death as a result of bleeding. Included trials had a low risk of bias in all domains (Supplementary file S4).

**Fig 2:**
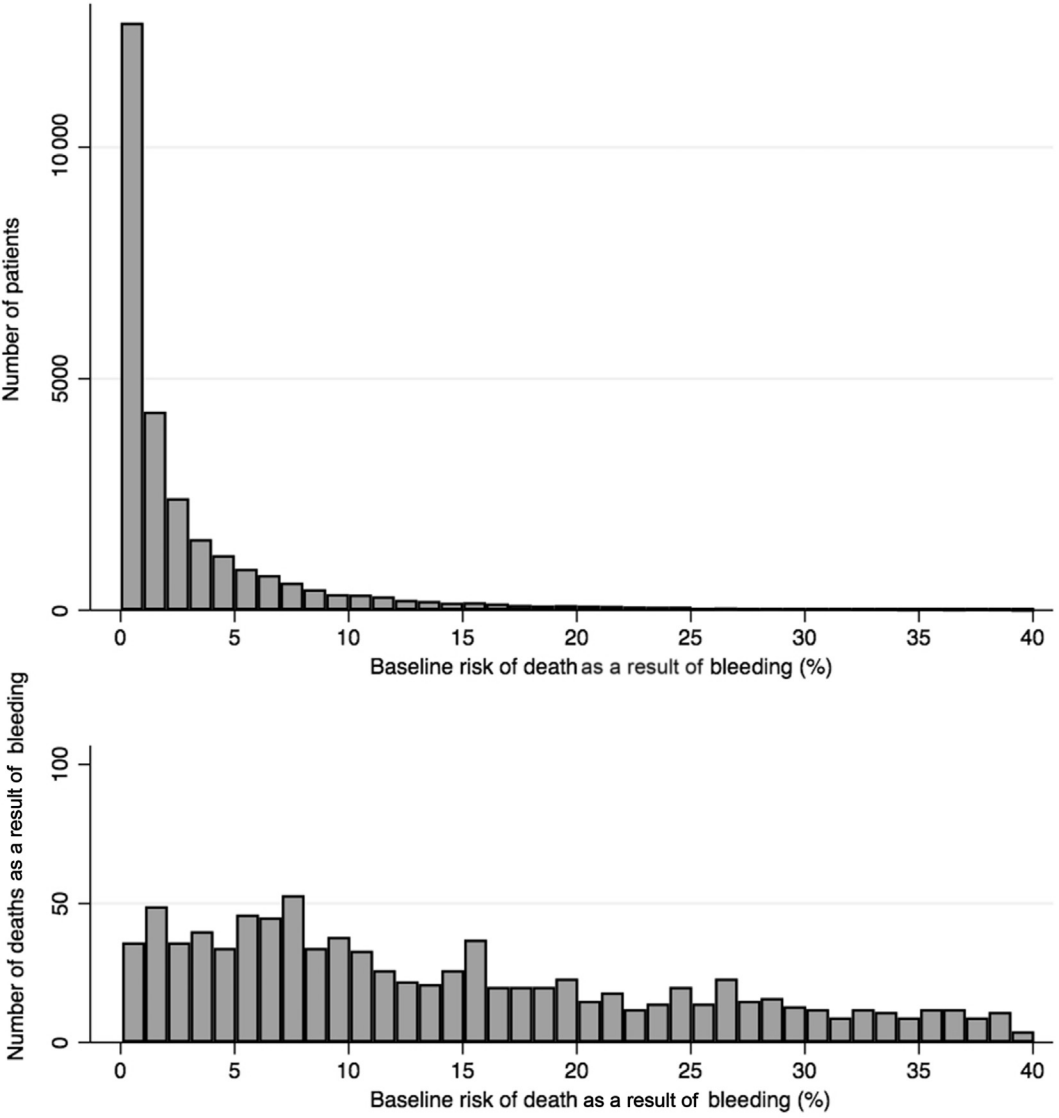
Number of patients and number of deaths according to baseline risk.

**Fig 3:**
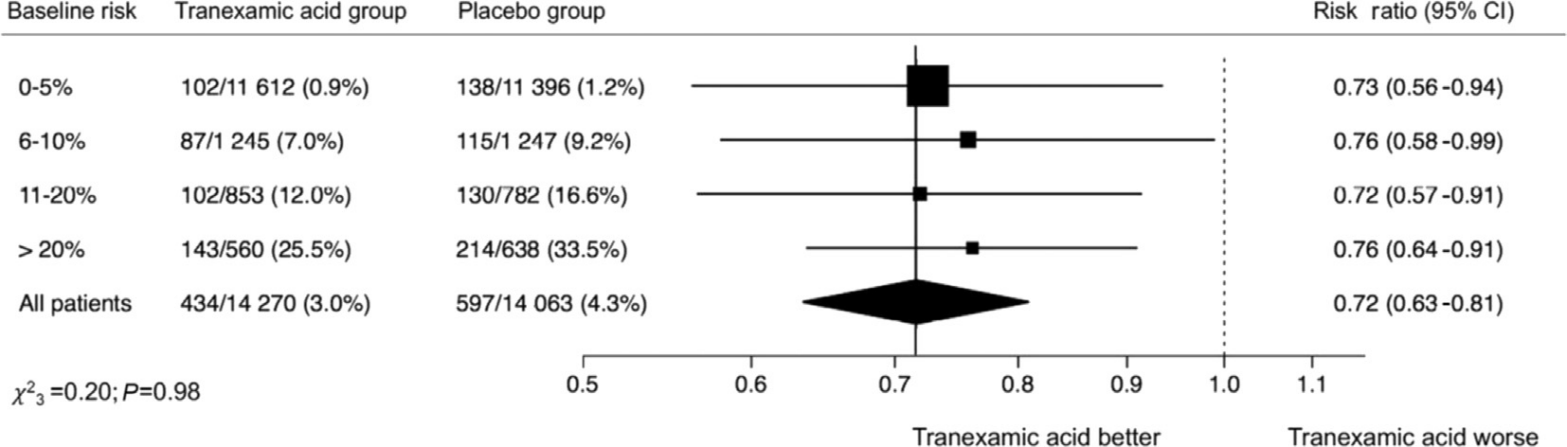
Effect of tranexamic acid on death as a result of bleeding by baseline risk. CI, confidence interval.

**Fig 4:**
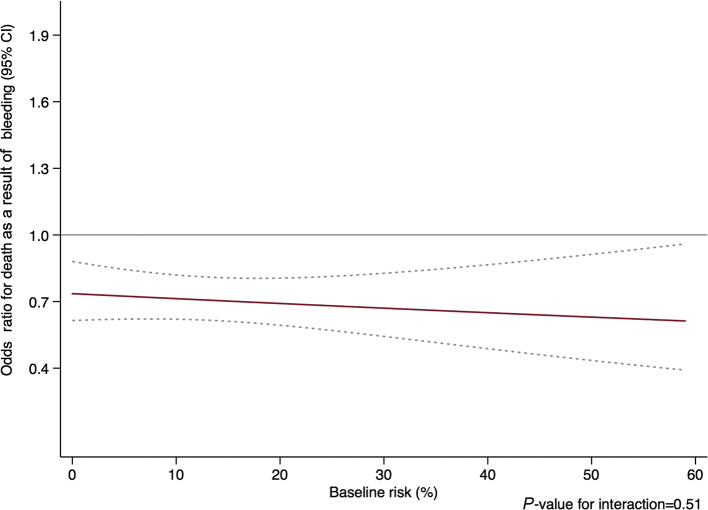
Effect of baseline risk on treatment benefit. CI, confidence interval.

We obtained individual patient data for 28 333 participants randomised within 3 h of the bleeding onset: 13 485 from the CRASH-2 trial and 14 848 from the WOMAN trial (Table 1). Of these, 14 270 participants received tranexamic acid and 14 067 received placebo. The baseline risk predictors for both models are detailed in the Supplementary file S5. The pooled discrimination of the prognostic models was good; C-statistic=0.88, 95% CI (0.87–0.89). The predicted risk was similar to the observed risk in the placebo group (ratio predicted/observed risk=1.00; 95% CI (0.92–1.07)) (Supplementary file S6). The baseline risk was higher in trauma patients than in women with postpartum haemorrhage. Most patients had a baseline risk under 5% (Fig. 2). Deaths as a result of bleeding occurred in all baseline risk categories with almost the same number of deaths as a result of bleeding. We reported 240 (1%), 202 (8%), 232 (14%), and 357 (30%) deaths in the low-, intermediate-, high-, and very high-risk categories, respectively. Deaths as a result of bleeding occurred in all categories of blood loss among women with postpartum haemorrhage (Supplementary file S7). The effect of tranexamic acid did not vary between trials (model 1: *P*=0.82). We found no heterogeneity in the interaction between treatment effect, baseline risk, and time to treatment (model 2: *P*=0.62 for the triple interaction). We did not find any significant interaction between the effect of tranexamic acid on death as a result of bleeding and baseline risk (model 3: *P*=0.51). Figure 3 shows crude risk ratios by categories of baseline risk. The treatment effect did not vary by baseline risk (Fig. 4). The risk of vascular occlusive events was similar according to baseline risk categories (Table 2). There was no increase in fatal and non-fatal occlusive events with tranexamic acid in any of the baseline risk categories (Supplementary file S8).

**Table 1:** Baseline characteristics of patients in participating trials.

	CRASH-2 trial (*n*=13 485)	Woman trial (*n*=14 848)	Total (*n*=28 333)
Predicted baseline risk, *n* (%)			
0–5	9063 (67.2)	13 945 (93.9)	23 008 (81.2)
6–10	2011 (14.9)	481 (3.2)	2492 (8.8)
11–20	1373 (10.2)	262 (1.8)	1635 (5.8)
>20	1038 (7.7)	160 (1.1)	1198 (4.2)
Missing	0 (0.0)	0 (0.0)	0 (0.0)
Mean baseline risk (sd)	6.9 (9.5)	1.6 (4.4)	4.1 (7.7)
Median baseline risk (IQR)	3.3 (1.4–7.9)	0.4 (0.1–1.3)	1.3 (0.3–4.2)
Age (yr), *n* (%)			
<25	3840 (28.5)	3973 (26.8)	7813 (27.6)
25–29	2400 (17.8)	4590 (30.9)	6990 (24.7)
30–34	1792 (13.3)	3802 (25.6)	5594 (19.8)
≥35	5453 (40.4)	2478 (16.7)	7931 (28.0)
Missing	0 (0.0)	5 (0.0)	5 (0.0)
Mean age (sd)	34.1 (14.0)	28.4 (5.7)	31.1 (10.9)
Median age (IQR)	30 (24–42)	28 (24–32)	29 (24–35)
Systolic BP (mm Hg), *n* (%)			
<75	2074 (15.7)	1011 (6.8)	3085 (11.0)
75–89	2360 (17.8)	1563 (10.5)	3923 (14.0)
≥90	8813 (66.5)	12 269 (82.7)	21 082 (75.1)
Missing	238 (1.8)	5 (0.0)	243 (0.9)
Mean systolic BP (sd)	96.6 (25.3)	101.5 (21.4)	99.2 (23.5)
Median systolic BP (IQR)	90 (80–110)	100 (90–110)	100 (90–110)
Time to treatment (h), *n* (%)			
≤1	7452 (55.3)	9220 (62.1)	16 672 (58.8)
1–3	6033 (44.7)	5628 (37.9)	11 661 (41.2)
Missing	0 (0.0%)	0 (0.0%)	0 (0.0%)
Mean time to treatment (sd)	1.5 (0.8)	1.0 (0.8)	1.3 (0.8)
Median time to treatment (IQR)	1 (1–2)	0.7 (0.4–1.5)	1 (0.5–2)

IQR, inter-quartile range; sd, standard deviation.

**Table 2:** Vascular occlusive events by treatment allocation according to baseline risk.

Baseline risk, *n* (%)	0–5%		6–10%		11–20%		>20%		*P*-value
	Tranexamic acid *N*=11 612	Placebo *N*=11 396	Tranexamic acid *N*=1245	Placebo *N*=1247	Tranexamic acid *N*=853	Placebo *N*=782	Tranexamic acid *N*=560	Placebo *N*=638	
Any vascular occlusive events	64 (0.6)	65 (0.6)	17 (1.4)	22 (1.8)	23 (2.7)	38 (4.9)	14 (2.7)	27 (4.2)	0.255
Fatal occlusive events	16 (0.1)	15 (0.1)	6 (0.5)	4 (0.3)	4 (0.5)	14 (1.8)	1 (0.2)	7 (1.1)	0.058
Myocardial infarction∗	8 (0.1)	14 (0.1)	3 (0.2)	7 (0.6)	6 (0.7)	13 (1.7)	7 (1.3)	12 (1.9)	0.909
Stroke∗	19 (0.2)	14 (0.1)	3 (0.2)	6 (0.5)	6 (0.7)	15 (1.9)	4 (0.7)	7 (1.1)	0.152
Pulmonary embolism∗	28 (0.2)	23 (0.2)	6 (0.5)	8 (0.6)	14 (1.6)	16 (2.1)	6 (1.1)	9 (1.4)	0.739
Deep vein thrombosis∗	12 (0.1)	19 (0.2)	7 (0.6)	2 (0.2)	6 (0.7)	4 (0.5)	3 (0.5)	5 (0.8)	0.214

∗ Includes both fatal and non-fatal events.

## Discussion

### Main findings

Our results show that many deaths from bleeding are in patients at low or intermediate risk and that the mortality reduction from tranexamic acid does not vary by baseline risk. We found no evidence of any increase in vascular occlusive events in any of the risk categories. Our study has important strengths and some limitations. First, we selected only randomised trials with more than 1000 patients to reduce selection bias. Small trials contribute very little evidence but could increase the risk of selection bias.^29^ Second, we used a rigorous method to develop prognostic models to predict baseline risk.^30^ Specifically, baseline risk was estimated using the entire dataset and not just the placebo group. By increasing the sample size and constraining the treatment effect in the regression equation, it improves both the precision of prediction and the calibration.^31^ Third, we performed logistic regression with baseline risk as a continuous variable since an on–off step function is biologically implausible. There was no interaction between treatment effect, trial, and time to treatment. Even though we restricted our analyses to patients treated within 3 h of bleeding onset, as recommended in clinical practice, we included trial and time to treatment in the model to avoid any residual confounding. Fourth, there were no missing outcome data and very few missing data for predictors of baseline risk (<1%). Nevertheless, we performed multiple imputation and used the whole dataset for analysis. We cannot exclude some measurement error in the predictors used to estimate the baseline risk and this could lead to regression dilution bias and over- or under-prediction in some patients.^32^ Misclassification of death as a result of bleeding is also possible, as death from thrombotic disseminated intravascular coagulation could be confused with death from bleeding. We cannot exclude some misclassification as a result of optimism of the model affecting calibration. We are reassured that optimism was low in the model developed for trauma and the selection of a limited number of predictors limits overfitting.^10 11^ Finally, the large sample size with more than 28 000 patients with acute bleeding treated within 3 h of onset gives precise results. However, estimates of the effects on adverse events are much less precise. The study included data from 38 countries across several continents and so the results should be widely generalisable to patients presenting to hospitals with postpartum haemorrhage and to trauma patients with, or at risk of, significant haemorrhage.

### Implications of the study

The main clinical implication of these results is that tranexamic acid treatment should be considered as an early preventive measure rather than a treatment for severe coagulopathic bleeding. Because of the large number of patients in the low- and intermediate-risk groups, these groups contribute a large number of bleeding deaths. Indeed, about one-quarter of deaths from bleeding occurred in patients who initially appeared to have a low risk of death. Early identification of bleeding can be challenging, especially in trauma. Patients without obvious bleeding sometimes have concealed bleeding and can suddenly deteriorate. Although early identification of bleeding by a CT scan or FAST (Focused Assement with Sonography for Trauma) vel is a priority, a definitive diagnosis can take up to 1 h, even in the best trauma systems. Hence, many major trauma patients without clinically apparent bleeding will not receive tranexamic acid soon enough unless early treatment is given to all major trauma patients whatever their apparent risk. Major trauma is usually defined as an injury or a combination of injuries that are potentially life-threatening or could lead to long-term disability. Because the full extent of the patient's injuries is unknown at initial assessment, trauma team activation criteria represent a pragmatic alternative definition of major trauma in the prehospital setting. As for obstetric bleeding, WHO guidelines recommend tranexamic acid in addition to standard care for all women with clinically diagnosed postpartum haemorrhage. However, if ‘in addition to’ is taken to mean that tranexamic acid should be given after standard care has been found to be insufficient to stop the bleeding, this will result in unnecessary treatment delay. Instead, we believe that early tranexamic acid treatment should be considered integral to standard care.

### Future studies

We found 13 ongoing trials of antifibrinolytic drugs in acute severe bleeding. Three of these could provide additional data on the treatment effect by baseline risk in extracranial bleeding. However, these ongoing trials are small and their inclusion is very unlikely to change our conclusions. However, additional trials could increase the power to detect adverse effects. Further individual patient level data meta-analyses that consider vascular occlusive events are needed.

## Conclusions

Tranexamic acid appears to be safe and effective regardless of the baseline risk for patients treated within 3 h since injury. Because many deaths are in patients at low and intermediate risk, tranexamic acid use should not be restricted to the most severely injured or bleeding patients. As tranexamic acid is safe, it should be considered as an early preventive measure rather than a treatment for severe coagulopathic bleeding.

## Authors' contributions

Designed the study: FXA, AGA, KK, IR

Designed and monitored the data collection from which this paper was developed: FXA, HSS, KK, IR

Analysed the data: FXA, AGA

Gave feedback about the clinical use: FXA, AGA

Wrote the first draft: FXA, IR

Contributed to writing and revising the paper: all authors

## Declaration of interest

The authors declare that they have no conflicts of interest.

## Funding

10.13039/100004440Wellcome Trust (grant 208870 to IR and HSS).
