# Supplementary material for: Effect of tranexamic acid by baseline risk of death in acute bleeding patients: a meta-analysis of individual patient-level data from 28 333 patients
Source: Br J Anaesth. 2020 Mar 19;124(6):676–83. doi: 10.1016/j.bja.2020.01.020 (PMC7271662; doi:10.1016/j.bja.2020.01.020)

Supplementary material

Supplementary method S1. MEDLINE search strategy.

Supplementary method S2. Equations of the different models.

Supplementary method S3. Characteristics of included and ongoing trials.

Supplementary method S4. Results of risk of bias assessment.

Supplementary method S5. Prognosis model to estimate baseline risk of death due to bleeding.

Supplementary figure S6. Performance of prognosis model predicting baseline risk of death due to bleeding.

Supplementary figure S7. Frequency of women with post-partum hemorrhage and death due to bleeding according to blood loss.

Supplementary table S8. Vascular occlusive events (fatal and non-fatal) by trial and overall.

Supplementary table S9. Sensitivity analysis with baseline risk estimate based on models developed with placebo arm only.

Supplementary Figure S10. Sensitivity analysis with baseline risk estimate based on models developed with placebo arm only: effect of baseline risk on treatment benefit.

Supplementary method S1. MEDLINE search strategy.

The searches used to identify trials for this study were run to 1 July 2018 and were not restricted by date, language or publication status.
Database: Ovid MEDLINE(R) <1946 to March Week 26 2018>

1 exp Antifibrinolytic Agents/
2 (anti-fibrinolytic* or antifibrinolytic* or antifibrinolysin* or anti-fibrinolysin* or antiplasmin* or anti-plasmin* or ((plasmin or fibrinolysis) adj3 inhibitor*)).ab,ti.
3 exp Aprotinin/
4 (Aprotinin* or kallikrein-trypsin inactivator* or bovine kunitz pancreatic trypsin inhibitor* or bovine pancreatic trypsin inhibitor* or basic pancreatic trypsin inhibitor* or BPTI or contrykal or kontrykal or kontrikal or contrical or dilmintal or iniprol or zymofren or traskolan or antilysin or pulmin or amicar or caprocid or epsamon or epsikapron or antilysin or iniprol or kontrikal or kontrykal or pulmin* or Trasylol or Antilysin Spofa or rp?9921 or antagosan or antilysin or antilysine or apronitin* or apronitrine or bayer a?128 or bovine pancreatic secretory trypsin inhibitor* or contrycal or frey inhibitor* or gordox or kallikrein trypsin inhibitor* or kazal type trypsin inhibitor* or (Kunitz adj3 inhibitor*) or midran or (pancrea* adj2 antitrypsin) or (pancrea* adj2 trypsin inhibitor*) or riker?52g or rp?9921or tracylol or trascolan or trasilol or traskolan or trazylol or zymofren or zymophren).ab,ti.
5 exp Tranexamic Acid/
6 (tranexamic or Cyclohexanecarboxylic Acid* or Methylamine* or amcha or trans-4-aminomethyl-cyclohexanecarboxylic acid* or t-amcha or amca or kabi 2161 or transamin* or exacyl or amchafibrin or anvitoff or spotof or cyklokapron or ugurol oramino methylcyclohexane carboxylate or aminomethylcyclohexanecarbonic acid or aminomethylcyclohexanecarboxylic acid or AMCHA or amchafibrin or amikapron or aminomethyl cyclohexane carboxylic acid or aminomethyl cyclohexanecarboxylic acid or aminomethylcyclohexane carbonic acid or aminomethylcyclohexane carboxylic acid or aminomethylcyclohexanecarbonic acid or aminomethylcyclohexanecarboxylic acid or aminomethylcyclohexanocarboxylic acid or aminomethylcyclohexanoic acid or amstat or anvitoff or cl?65336 or cl65336 or cyclocapron or cyclokapron or cyklocapron or exacyl or frenolyse or hexacapron or hexakapron or tranex or TXA).ab,ti.
7 exp Aminocaproic Acids/ or exp 6-Aminocaproic Acid/
8 (((aminocaproic or amino?caproic or aminohexanoic or amino?hexanoic or epsilon-aminocaproic or E-aminocaproic) adj2 acid*) or epsikapron or cy-116 or cy116 or epsamon or amicar or caprocid or lederle or Aminocaproic or aminohexanoic or amino caproic or amino n hexanoic or acikaprin or afibrin or capracid or capramol or caprogel or caprolest or caprolisine or caprolysin or capromol or cl 10304 or EACA or eaca roche or ecapron or ekaprol or epsamon or epsicapron or epsilcapramin or epsilon amino caproate or epsilon aminocaproate or epsilonaminocaproic or etha?aminocaproic or ethaaminocaproich or emocaprol or hepin or ipsilon or jd?177or neocaprol or nsc?26154 or tachostyptan).ab,ti.
9 exp 4-Aminobenzoic Acid/tu [Therapeutic Use]
10 (PAMBA or para-aminomethylbenzoic or p-aminomethylbenzoic or amino?methylbenzoic acid or Gumbix or Styptopur or H-4-AMB-OH or CAS:56-91-7 or H-4AMBZ-OH or NH2-CH2-PH4-COOH or TIMTEC-BB SBB006704 or "RARECHEM AL BW 0005" or Amino-p-toluicacid).ti,ab.
11 1 or 2 or 3 or 4 or 5 or 6 or 7 or 8 or 10
12 randomi?ed.ab,ti.
13 randomized controlled trial.pt.
14 controlled clinical trial.pt.
15 placebo.ab.
16 clinical trials as topic.sh.
17 randomly.ab.
18 trial.ti.
19 12 or 13 or 14 or 15 or 16 or 17 or 18
20 (animals not (humans and animals)).sh.
21 19 not 20
22 11 and 21

Supplementary method S2. Equations of the different models.

1) Logistic regression assessing overall treatment effect and homogeneity of treatment effect across trials

Logit (p(Y = 1)) = $\beta_0 + \beta_1$ S + β_2 X + β_3 (X*S) [model-1]

With Y = 1, the outcome did not die from bleeding for patient i in trial j, S is the trial (CRASH-2 S=0, WOMAN S=1), X is treatment (tranexamic acid is X=1, placebo is X=0).
Then β_0 is the log(odds) in the placebo group in the CRASH-2 trial, β_1 is the difference between trials in placebo group, β_2 the effect of tranexamic acid in CRASH-2 trial, and β_3 is the interaction between treatment effect and trial.

2) Logistic regression estimating non-linear effect of intervention by baseline risk and its interaction with time to treatment (triple interaction).

Logit (p(Y = 1)) = $\beta_0 + \beta_1$ T + β_2 X + β_3 BR + β_4 (X*T) + β_5 (BR*T) + β_6 (BR*X) + β_7 (BR*X*T) [model-2]

With Y, X coded as in [model-1]. T is the time to treatment in hours. BR is the baseline risk.
Then β_0 is the log(odds) in the placebo group when T=0 and BR=0; β_1 is the linear effect of time to treatment in the placebo group at BR=0; β_2 the effect of tranexamic acid at T=0 and BR=0; β_3 is the linear effect of baseline risk in the placebo group at T=0; β_4 is the interaction between treatment effect and time to treatment at BR=0; β_5 is the interaction between time to treatment and baseline risk in the placebo group; β_6 is the interaction of baseline risk with the treatment at T=0; β_7 is the triple interaction of baseline risk with the treatment and the time to treatment.

3) Logistic regression estimating linear effect of intervention by baseline risk (we assume this interaction is the same in both trials).

Logit (p(Y = 1)) = $\beta_0 + \beta_1$ S + β_2 X + β_3 BR + β_4 (BR*X) + β_5 T [model-3]

With Y, S, X, T, BR coded as in [model-1] and [model-2];
Then, β_0 is the log(odds) in the placebo group in the CRASH-2 trial when BR=0; β_1 is the difference between trials; β_2 is the effect of tranexamic when BR=0;; $\beta_3$is the linear effect of baseline risk in the placebo group of both trials ; β_4 is the interaction of baseline risk with the treatment; β_5 is the effect of time to treatment.

Supplementary method S3. Characteristics of included and ongoing trials.

Trial ID	Title	Participants	Intervention	Outcomes
Included trials				
CRASH-2[1]	A large randomised placebo controlled trial among trauma patients with, or at risk of, significant haemorrhage, of the effects of anti-fibrinolytic treatment on death and transfusion requirement.	N=20,211<br><br>Adult (>16 years) trauma patients with, or at risk of, significant bleeding.	A loading dose of 1 g tranexamic acid or placebo will be administered as soon possible, followed by a maintenance dose of 1 g TXA or placebo over eight hours.	Primary: Death.<br>Secondary: Vascular occlusive events, blood transfusion requirements, disability.
WOMAN[2]	Tranexamic acid for the treatment of postpartum haemorrhage: an international randomised, double blind, placebo controlled trial	N=20,060<br><br>Women with clinically diagnosed postpartum haemorrhage following vaginal delivery of a baby or caesarean section. The clinical diagnosis of PPH may be based on any of the following: estimated blood loss after vaginal delivery of a baby> 500 mL OR >1000 mL from caesarean section OR blood loss sufficient to compromise the haemodynamic status of the woman.	1g of T tranexamic acid by intravenous injection or placebo (sodium chloride 0.9%) given as soon as possible after randomisation.<br>If after 30 minutes bleeding continues, or if it stops and restarts within 24 hours after the first dose, a second dose may be given.	Primary: Death or hysterectomy.<br>Secondary: Death, surgical intervention, blood transfusion, health status, thromboembolic events, other relevant medical events, length of stay at hospital/time spent at an intensive care unit, mechanical ventilation, status of breastfed baby/ies.
TICH-2[3]	Tranexamic acid for hyperacute primary IntraCerebral Haemorrhage	N=2325<br>Adult patients with acute primary intracerebral haemorrhage within 8 hours of stroke onset.	Tranexamic acid 1 g or placebo in 100 ml sodium chloride 0.9% infusion bag intravenously as a loading dose infusion over 10 min, followed by infusion of tranexamic<br>acid 1 g or placebo in 250 ml sodium chloride 0.9%<br>infusion bag over 8 h.	Primary: Death or dependency at day 90<br>Secondary: Neurological impairment at day 7 or discharge if sooner, disability (Barthel index) at day 90, Quality of Life (EuroQol) at day 90, cognition at day 90, costs: length of stay in hospital, re-admission, institutionalisation, radiological efficacy/safety (CT scan): change in haematoma volume from baseline to day 2, haematoma location and new infarction.
Excluded Trials				
ATACAS[4]	Aspirin and tranexamic acid for Coronary Artery Surgery Trial	N=4662<br><br>Adults undergoing coronary-artery surgery and at risk of perioperative complications.	Tranexamic acid (100mg/kg) or saline administered 30 minutes after induction of anaesthesia (dose of tranexamic acid halved to 50mg after 1392 patients enrolled)	Primary: Composite outcome of all-cause 30 day mortality or thrombotic event<br>Secondary: Death, nonfatal myocardial infarction, pulmonary embolism, stroke, acute renal failure, bowel infarction), reoperation due to major haemorrhage or cardiac tamponade, blood transfusion.
TRAAP[5]	Tranexamic acid for Preventing Postpartum Haemorrhage Following a Vaginal Delivery: a Multicenter Randomised Double Blind Placebo Controlled Trial	N = 4079<br><br>Women in labour for a planned vaginal singleton delivery, at a term ≥ 35 weeks.	1g tranexamic acid or placebo will be administered intravenously just after birth.	Primary: incidence of PPH, defined by blood loss ≥500 mL<br>Secondary: Mean blood loss at 15 minutes after birth; mean total blood loss; incidence of severe PPH; need for supplementary uterotonic treatment;

				postpartum transfusion; need for invasive second-line procedures for PPH; haemoglobin, hematocrit; hemodynamic tolerance; mild adverse effects; tolerance lab tests; severe adverse effects
Ongoing trials				
CRASH-3[6] (ISRCTN15088122)<br><br>Expected completion date: December 2018	Tranexamic acid for the treatment of significant traumatic brain injury: an international randomised, double blind placebo controlled trial	N=13,000 (target)<br><br>Adults with traumatic brain injury, who are within eight hours of injury, with any intracranial bleeding on CT scan or who have a GCS of 12 or less, and have no significant extra-cranial haemorrhage.	Loading dose of tranexamic acid (1 gram by intravenous injection) or placebo (sodium chloride 0.9%) given as soon as possible after randomisation. Maintenance dose of tranexamic acid (1 gram by intravenous injection) or placebo (sodium chloride 0.9%) given after the loading dose is finished.	Primary: death in hospital within 28 days of injury.<br>Secondary: vascular occlusive events, disability, seizures, neurosurgical intervention, days in intensive care, other adverse events.
HALT-IT[7] (ISRCTN11225767)<br><br>Expected completion date: October 2017	Tranexamic acid for the treatment of gastrointestinal haemorrhage: an international randomised, double blind placebo controlled trial	N=8000 (target)<br><br>Adults with acute significant upper or lower gastrointestinal bleeding.	Loading dose of tranexamic acid (1g by intravenous injection) or placebo (sodium chloride 0.9%) will be given as soon as possible after randomisation, followed by an intravenous infusion of 3g of tranexamic acid or placebo (sodium chloride 0.9%) over 24 hours.	Primary: death in hospital (cause-specific mortality will also be recorded)<br>Secondary: Re-bleeding, need for salvage surgery or radiological intervention, blood transfusion, thromboembolic events, other adverse medical events, functional status, time spent at an intensive care unit, length of stay in hospital
Shanghai FMIH-TXA1[8] NCT02936661<br><br>Expected completion date: March 2019	Tranexamic acid for Preventing Postpartum Hemorrhage After Cesarean Section	N=6700 (target)<br><br>Women giving birth by cesarean section.	Tranexamic acid or placebo	Primary: postpartum haemorrhage<br>Secondary: the amount of postpartum bleeding
PATCH[9] NCT02187120<br><br>Expected completion date: January 2021	A Multi-centre Randomised, Double-blinded, Placebo-controlled Trial of Pre-hospital Treatment With tranexamic acid for Severely Injured Patients at Risk of Acute Traumatic Coagulopathy.	N= 1184 (target)<br><br>Adult patients (age ≥18 years); injured through any mechanism; COAST score≥3.	1g tranexamic acid or placebo (0.9% NaCl) by slow intravenous injection as early as possible following injury. Soon after arrival to the emergency department, patients will be given 1g tranexamic acid or placebo infused intravenously for 8 hours.	Primary: Favourable outcome at six months (moderate disability to good recovery, GOSE scores 5-8) compared to those who have died (GOSE 1), or have severe disability (GOSE 2-4).<br>Secondary: Units of blood products used in the first 24 hours; coagulation profile; ICU ventilator-free days in first 28 days; vascular occlusive events; mortality; proportion of deaths due to: bleeding, vascular occlusion, multi-organ failure and head injury; cumulative incidence of sepsis at 28 days or hospital discharge whichever occurs first; severity of chronic pain 6 months after injury and its interference with daily activities measured using the modified Brief Pain Inventory; Quality of life (SF12® and EQ5D) at 6 months.

STAAMP[10]<br>NCT02086500<br><br>Expected completion date: March 2019	Study of tranexamic acid During Air Medical Prehospital Transport Trial For Trauma Patients At Risk Of Hemorrhage	N=1000 (target)<br><br>Adult (18-90 years) trauma patients within 2 hours of injury.<br><br>Setting: USA	1g tranexamic acid or placebo during air medical transport.	Primary outcome: 30 day mortality.<br>Secondary outcomes: hyperfibrinolysis, acute lung injury, multiple organ failure, nosocomial infection, mortality, early seizures, pulmonary embolism, early resuscitation needs, early coagulopathy as measured by INR and rapid thromboelastography parameters, early inflammatory response, plasmin levels, leukocyte, platelet and complement activation.
NCT03364491[11] (MFMU Network)<br><br>Expected completion date: December 2020	Tranexamic Acid for the Prevention of Obstetrical Hemorrhage After Cesarean	N=11000 (target)<br><br>Women giving birth by scheduled or unscheduled cesarean section<br><br>Setting: USA	1g tranexamic acid or placebo	Primary outcome: Maternal death or transfusion of 1 or more units of packed red blood cells (up to hospital discharge or 7 days)<br>Secondary outcome: Blood loss, composite surgical or radiological intervention to control bleeding, composite maternal death and thromboembolic events, transfusion related acute lung injury, transfusion of other blood products, transfusion of more than 4 RBC, acute kidney injury, thromboembolic events, seizure, ingfection, admission to ICU, change in haemoglobin, TXA side-effects, length of stay, hospital re-admission, transfusion reaction
NCT01990768[12]<br><br>Expected completion date: January 2019	Prehospital Tranexamic Acid Use for Traumatic Brain Injury	N=1002 (target) 967 recruited<br><br>Moderate to severe TBI (GCS score ≤12)<br><br>Setting: Prehospital, Canada, USA	1g tranexamic acid prior to hospital arrival followed by a 1g infusion or 2g tranexamic acid prior to admission or placebo	Primary outcome: Glasgow Outcome Scale Extended (GOS-e) at 6 months.<br>Secondary outcome: Death at 28 days, disability rating scale at discharge and 6 months, Unfavourable outcome Dichotomized GOS-e, Number ICH, Marshall score CT, Rotterdam score CT, Neurosurgical intervention, Hospital free-days, ICU free-days, seizure, thromboembolic event (CVD, DVT, MI, PE).
TRAAP-2[13]<br>NCT03431805<br><br>Expected completion date: June 2020	Tranexamic acid for Preventing Postpartum Haemorrhage Following a Cesarean Delivery	N=4524 (target)<br><br>Women admitted for caesarean delivery before or during labor (term≥34)<br><br>Setting: France	1g tranexamic acid or placebo with prophylactic uterotonic 3 minutes after birth.	Primary outcome: incidence of PPH, defined by blood loss >1000 mL at day 2.<br>Secondary outcome: blood loss >500; >1500, mean blood loss, incidence of transfusion, mean RBC transfused, incidence embolization or surgery, change in haemoglobin, HR, SBP, DBP, nausea, vomiting, phosphenes, dizziness, vreat, urea, prothrombin, asat, alat,

				bilirubin, fibrinogen, DVP, PE, MI, any thrombotic event, seizure, women's satisfaction,m shock, ICU, death from any cause
WOMAN-2[14]<br>NCT03475342<br><br>Expected completion date: March 2022	World Maternal Antifibrinolytic Trial 2	N=10000 (target)<br><br>Women with moderate or severe anemia (Hb<100g/L or packed cell volume <30%) planned to give birth vaginally<br><br>Setting: International	1g tranexamic acid or placebo administered at delivery (no later than 15 minutes after umbilical cord is clamped)	Primary outcome: PPH at 24H(blood loss>500 or any blood loss sufficient to compromise haemodynamic stability).<br>Secondary outcome: blood loss, Hb, Haemodynamic instability, shock index, quality of life (maternal), side-effects, exercise tolerance, intervention for control PPH, blood transfusion, vascular occlusive events, anemia, organ dysfunction, in-hospital death, length of hospital stay, transfer to higher facility, status baby, thrombotic events in breastfed babies, adverse events.
POISE-3 trial[15]<br>NCT03505723<br><br>Expected completion date: December 2022	PeriOperative ISchemic Evaluation-3 Trial	N=10000 (target)<br><br>Patent undergoing noncardiac surgery with ≥ 45 years of age and expected to require at least an overnight hospital admission after surgery.<br><br>Setting: International		Primary outcome : A composite of life-threatening bleeding, major bleeding, and critical organ bleeding at 30 days.<br>A composite of myocardial infarction, non-hemorrhagic stroke, peripheral arterial thrombosis, and symptomatic proximal venous thromboembolism at 30 days.<br>For patients in the blood pressure management arm: A composite of vascular death, and non-fatal myocardial infarction, stroke, and cardiac arrest at 30 days.

References

1 CRASH-2 trial collaborators, Shakur H, Roberts I, *et al.* Effects of tranexamic acid on death, vascular occlusive events, and blood transfusion in trauma patients with significant haemorrhage (CRASH-2): a randomised, placebo-controlled trial. *Lancet Lond Engl* 2010; **376**: 23–32.

2 Effect of early tranexamic acid administration on mortality, hysterectomy, and other morbidities in women with post-partum haemorrhage (WOMAN): an international, randomised, double-blind, placebo-controlled trial. *Lancet Lond Engl* 2017; **389**: 2105–16.

3 Sprigg N, Flaherty K, Appleton JP, *et al.* Tranexamic acid for hyperacute primary IntraCerebral Haemorrhage (TICH-2): an international randomised, placebo-controlled, phase 3 superiority trial. *Lancet Lond Engl* 2018; **391**: 2107–15.

4 Myles PS, Smith JA, Forbes A, *et al.* Tranexamic Acid in Patients Undergoing Coronary-Artery Surgery. *N Engl J Med* 2017; **376**: 136–48.

5 Sentilhes L, Winer N, Azria E, *et al.* Tranexamic Acid for the Prevention of Blood Loss after Vaginal Delivery. *N Engl J Med* 2018; **379**: 731–42.

6 Dewan Y, Komolafe EO, Mejía-Mantilla JH, Perel P, Roberts I, Shakur H. CRASH-3 - tranexamic acid for the treatment of significant traumatic brain injury: study protocol for an international randomized, double-blind, placebo-controlled trial. *Trials* 2012; **13**: 87.

7 Roberts I, Coats T, Edwards P, *et al.* HALT-IT - tranexamic acid for the treatment of gastrointestinal bleeding: study protocol for a randomised controlled trial. *Trials* 2014; **15**. DOI:10.1186/1745-6215-15-450.

8 Shanghai First Maternity and Infant Hospital. Tranexamic Acid for Preventing Postpartum Hemorrhage After Cesarean Section (NCT02936661). https://clinicaltrials.gov/ct2/show/record/NCT02936661 (accessed April 3, 2019).

9 Mitra B, Mazur S, Cameron PA, *et al.* Tranexamic acid for trauma: filling the 'GAP' in evidence. *Emerg Med Australas EMA* 2014; **26**: 194–7.

10 Brown JB, Neal MD, Guyette FX, *et al.* Design of the Study of Tranexamic Acid during Air Medical Prehospital Transport (STAAMP) Trial: Addressing the Knowledge Gaps. *Prehospital Emerg Care Off J Natl Assoc EMS Physicians Natl Assoc State EMS Dir* 2015; **19**: 79–86.

11 Clifton, Rebecca. Tranexamic Acid for the Prevention of Obstetrical Hemorrhage After Cesarean. https://clinicaltrials.gov/ct2/show/NCT03364491.

12 May, Susanne. Prehospital Tranexamic Acid Use for Traumatic Brain Injury (NCT01990768). https://clinicaltrials.gov/ct2/show/record/NCT01990768?term=NCT01990768&rank=1 (accessed April 3, 2019).

13 Sentilhes, Loic. TRAnexamic Acid for Preventing Postpartum Hemorrhage Following a Cesarean Delivery :a Multicenter Randomised, Double Blind Placebo Controlled Trial (TRAAP2). https://clinicaltrials.gov/ct2/show/record/NCT03431805?term=TRAAP2&rank=1 (accessed April 3, 2019).

14 Ker K, Roberts I, Chaudhri R, *et al.* Tranexamic acid for the prevention of postpartum bleeding in women with anaemia: study protocol for an international, randomised, double-blind, placebo-controlled trial. *Trials* 2018; **19**. DOI:10.1186/s13063-018-3081-x.

15 Devereaux, PJ. PeriOperative ISchemic Evaluation-3 Trial (POISE-3). https://clinicaltrials.gov/ct2/show/study/NCT03505723?show_locs=Y#locn (accessed April 3, 2019).

Supplementary method S4. Results of risk of bias assessment.

CRASH-2

Domain	Judgement	Justification
Sequence generation	Low	Computer-generated.
Allocation concealment	Low	Tranexamic acid and placebo were packaged in identical ampoules. Recruiting hospitals with reliable telephone access used a telephone randomisation service, hospitals without, used a local pack system.
Blinding	Low	Participants, clinicians and trial staff were blinded to treatment allocation.
Incomplete outcome data	Low	Over 99% of patients were followed up and contributed outcome data.
Selective outcome reporting	Low	Prospectively registered and data on all pre-specified outcomes available for analysis.

WOMAN

Domain	Judgement	Justification
Sequence generation	Low	Computer-generated.
Allocation concealment	Low	Tranexamic acid and placebo were packed in sequentially numbered, sealed, treatment boxes.
Blinding	Low	Participants, clinicians and trial staff were blinded to treatment allocation.
Incomplete outcome data	Low	Over 99% of patients were followed up and contributed outcome data.
Selective outcome reporting	Low	Prospectively registered and data on all pre-specified outcomes available for analysis.

Supplementary method S5. Prognosis model to estimate baseline risk of death due to bleeding

CRASH-2 trial

$Pr = 1/(1+e^{-xb})$

$xb = 0.534 + RI + (0.061 * Age) – (1.4e^{-3} * Age^2) + (1.2e^{-05} * Age^3) + (0.023 * SBP) – (5.4e\text{-}04 * SBP^2) + (1.6e^{-06} * SBP^3) – (0.634 * GCS) + (0.074 * GCS^2) – (2.9e^{-3} * GCS^3) – (8.6e^{-3} * HR) + (1.0e\text{-}04 * HR^2) – (0.171 * RR) + (0.006 * RR^2) – (5.4e^{-05} * RR^3)$

RI: Random Intercept by country
Age (Year)
SBP: Systolic Blood Pressure (mmHg)
HR: Heart Rate (Beat per min)
RR: Respiratory Rate (Breath per minute)
GCS: Glasgow Coma Scale
Penetrating: Penetrating Injury

WOMAN trial

$Pr = 1/(1+e^{-xb})$

$xb = -8.66 + RI + (Age * 0.06) – (SBP * 0.01) – (SBP^2 * 3\ e^{-4}) + (SBP^3 * 1.6\ e^{-6}) + (BL * 2\ e^{-3}) – (BL^2 * 3\ e^{-7}) – (PP * 1.05) – (UA * 0.32) + (HI * 1.56) - (Delivery * 0.72)$

RI: Random Intercept by country
Age (Year)
SBP: Systolic Blood Pressure (mmHg)
BL: Blood Loss (ml)
PP: Placenta Previa (Yes=1, No=0)
UA: Uterine Atony (Yes=1, No=0)
HI: Haemodynamic instability (Yes=1, No=0)
Delivery: 0=Vaginal delivery; 1=caesarean section

Supplementary figure S6. Performance of prognosis model predicting baseline risk of death due to bleeding.

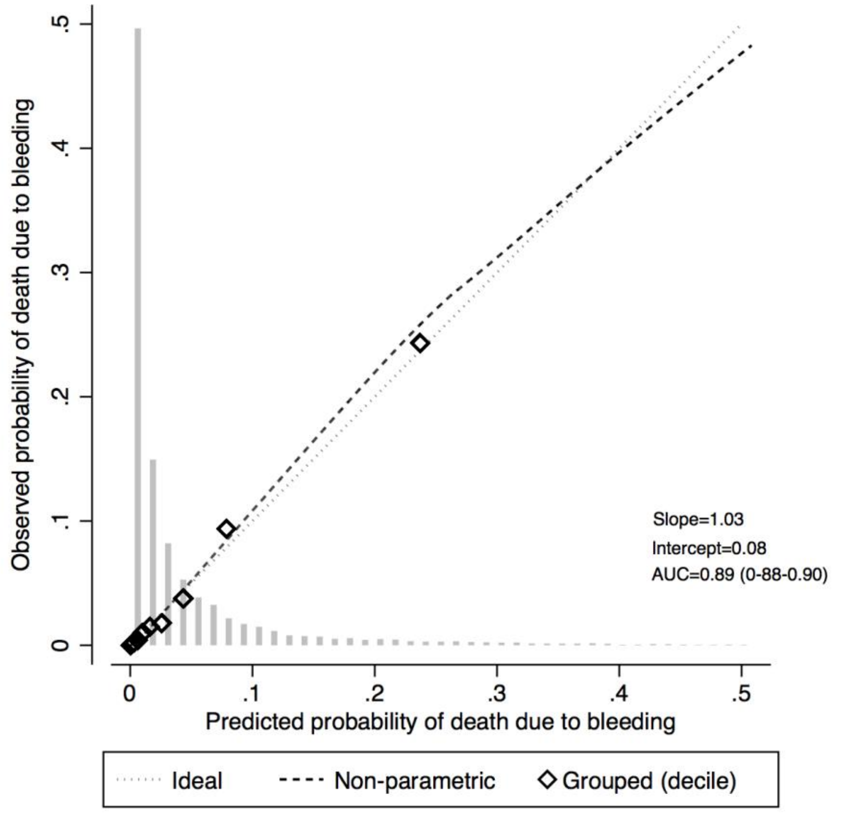

Supplementary figure S7. Frequency of women with post-partum hemorrhage and death due to bleeding according to blood loss.

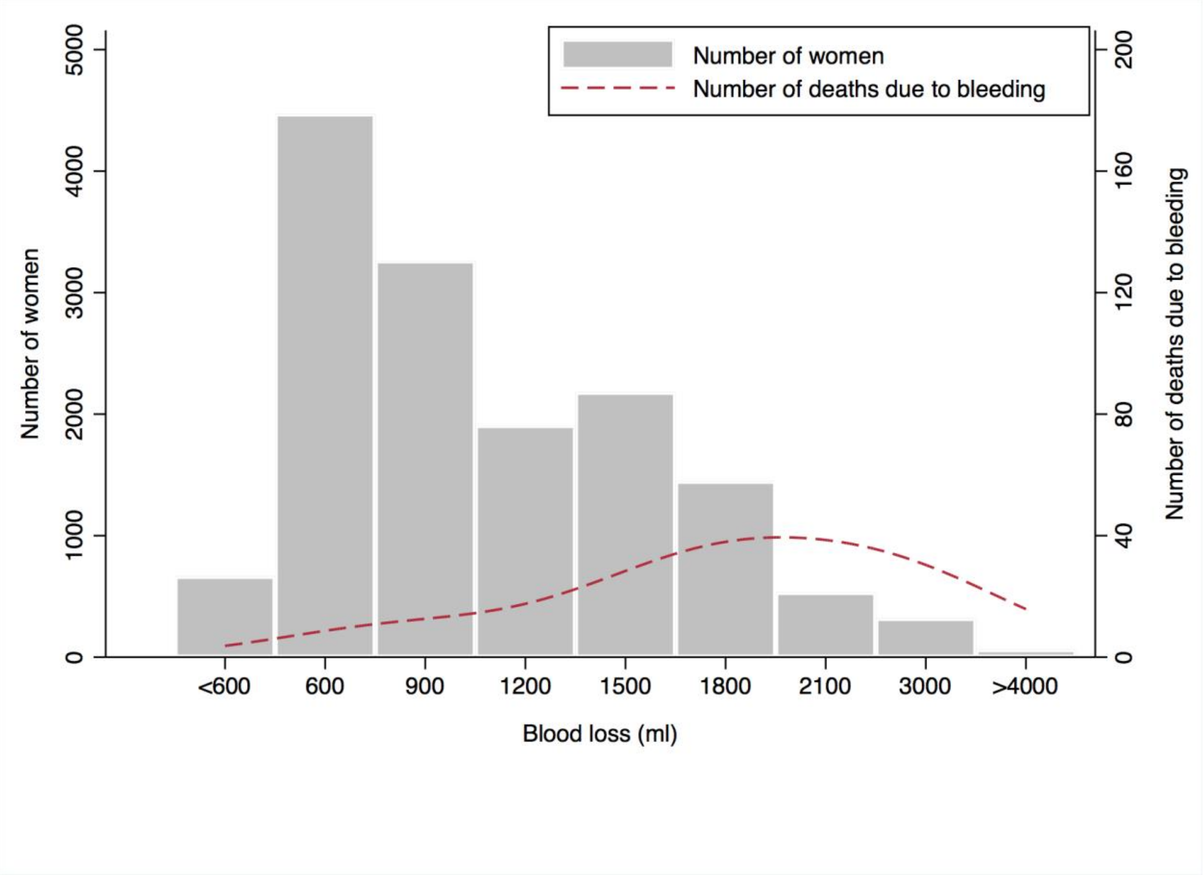

Supplementary table S8. Vascular occlusive events (fatal and non-fatal) by trial and overall.

	CRASH-2 trial			WOMAN-trial			Overall Trials		
Baseline risk	Tranexamic acid n (%)	Placebo n (%)	RR (95% CI)	Tranexamic acid n (%)	Placebo n (%)	RR (95% CI)	Tranexamic acid n (%)	Placebo n (%)	RR (95% CI)
0-5%	45 / 4587 (1.0)	55 / 4476 (1.2)	0.80 (0.54-1.18)	19 / 7003 (0.3)	10 / 6920 (0.1)	1.87 (0.87-4.02)	64 / 11612 (0.6%)	65 / 11396 (0.6%)	0.97 (0.69-1.36)
6-10%	15 / 988 (1.5)	22 / 1001 (2.2)	0.71 (0.36-1.35)	2 / 257 (0.8)	0 / 224 (0)	-	17 (1.4%)	22 (1.8%)	0.77 (0.41-1.45)
11-20%	22 / 731 (3.0)	36 / 642 (5.6)	0.54 (0.32-0.90)	1 / 122 (0.8)	2 / 140 (1.4)	0.57 (0.05-6.25)	23 (2.7%)	38 (4.9%)	0.55 (0.33-0.92)
>20%	13 / 478 (2.7)	25 / 560 (4.4)	0.61 (0.32-1.18)	1 / 82 (1.2)	2 / 78 (2.6)	0.48 (0.04-5.14)	14 (2.7%)	27 (4.2%)	0.59 (0.31-1.12)
Test for homogeneity, P Value			0.040			0.367			0.076

Supplementary table S9. Sensitivity analysis with baseline risk estimate based on models developed with placebo arm only.

	Main analysis (baseline risk based on both arm)				Sensitivity analysis (baseline risk based on placebo arm)			
	**Overall adjusted effect**	**P value**	**Test for homogeneity***	**P Value**	**Overall adjusted effect**	**P value**	**Test for homogeneity***	**P Value**
**Baseline risk by categories (RR)**	**0.74 (0.66-0.83)**	**<0.001**	**0.20**	**0.978**	**0.74 (0.66-0.83)**	**<0.001**	**4.44**	**0.218**
**Model 3 (interaction TXA-Baseline risk (OR)**	**0.74 (0.61-0.88)**	**0.001**	**-0.66**	**0.510**	**0.74 (0.62-0.89)**	**0.001**	**-1.03**	**0.305**

***by categories or for interaction**
TXA: Tranexamic acid; RR: Risk ratio; OR: Odds ratio

Supplementary Figure S10. Sensitivity analysis with baseline risk estimate based on models developed with placebo arm only: effect of baseline risk on treatment benefit.

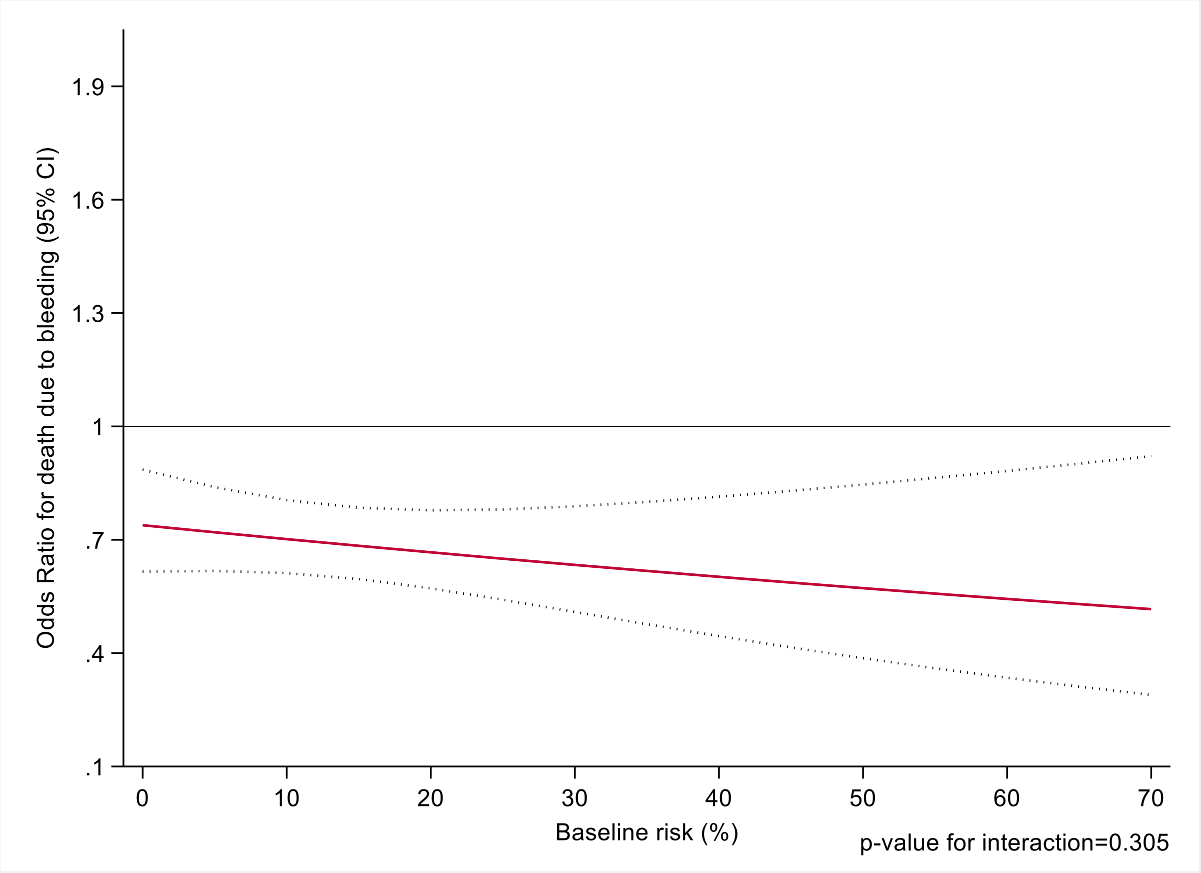

Supplement: Supplementary file 1 [file mmc1.pdf]
